# Contribution of the Environment, Epigenetic Mechanisms and Non-Coding RNAs in Psoriasis

**DOI:** 10.3390/biomedicines10081934

**Published:** 2022-08-09

**Authors:** Charalabos Antonatos, Katerina Grafanaki, Paschalia Asmenoudi, Panagiotis Xiropotamos, Paraskevi Nani, Georgios K. Georgakilas, Sophia Georgiou, Yiannis Vasilopoulos

**Affiliations:** 1 Laboratory of Genetics, Section of Genetics, Cell Biology and Development, Department of Biology, University of Patras, 26504 Patras, Greece; 2 Department of Dermatology, School of Medicine, University Hospital of Patras, University of Patras, 26504 Patras, Greece; 3 Laboratory of Hygiene and Epidemiology, Department of Clinical and Laboratory Research, Faculty of Medicine, University of Thessaly, 38334 Volos, Greece

**Keywords:** epigenetics, psoriasis, methylation, histone, ncRNAs

## Abstract

Despite the increasing research and clinical interest in the predisposition of psoriasis, a chronic inflammatory skin disease, the multitude of genetic and environmental factors involved in its pathogenesis remain unclear. This complexity is further exacerbated by the several cell types that are implicated in Psoriasis’s progression, including keratinocytes, melanocytes and various immune cell types. The observed interactions between the genetic substrate and the environment lead to epigenetic alterations that directly or indirectly affect gene expression. Changes in DNA methylation and histone modifications that alter DNA-binding site accessibility, as well as non-coding RNAs implicated in the post-transcriptional regulation, are mechanisms of gene transcriptional activity modification and therefore affect the pathways involved in the pathogenesis of Psoriasis. In this review, we summarize the research conducted on the environmental factors contributing to the disease onset, epigenetic modifications and non-coding RNAs exhibiting deregulation in Psoriasis, and we further categorize them based on the under-study cell types. We also assess the recent literature considering therapeutic applications targeting molecules that compromise the epigenome, as a way to suppress the inflammatory cutaneous cascade.

## 1. Introduction

**Figure 1:**
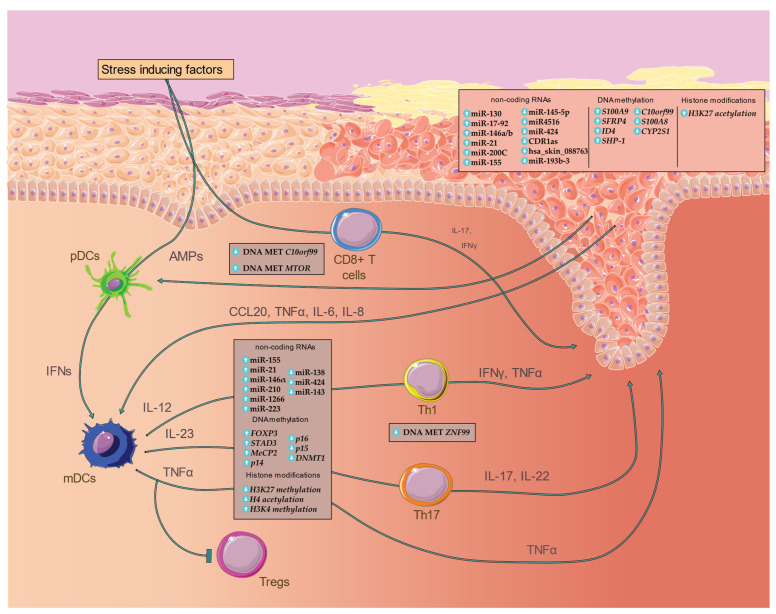
Overview of the inflammatory cascade observed in PsO, as well as key deregulated epigenetic factors and ncRNAs during the disease progression.

Psoriasis (PsO) is a chronic, inflammatory skin disease with its prevalence ranging from 1.83% to 5.32% in central European adults [1], with similar frequencies observed in white individuals in the United States [2]. PsO’s manifestation lies in the epidermal keratinocytes (KCs), where the perturbation of inflammatory and cell-cycle-related pathways leads to their uncontrolled proliferation, aberrant differentiation and the development of distinctive, erythematous plaques on the skin surface [3]. Significant progress has been accomplished in characterizing the mechanisms involved in the pathogenesis of PsO, which are related to the activation of immune cell types and the maintenance of the chronic inflammation through the production by KCs of numerous signaling and chemotactic molecules [3,4]. Specifically, antimicrobial peptides (AMPs) produced by KCs and melanocyte auto-antigens, such as ADAMTSL5, in response to cell damage and altered microbial environment stimulate Toll-like Receptor (TLR) 9 and 8 signaling pathways in the plasmacytoid dendritic cells (pDCs) and myeloid dendritic cells (mDCs), respectively [5,6]. The pDCs are activated and type I IFN is produced, inducing both the maturation of mDCs and the secretion of proinflammatory cytokines, such as IL-12, IL-23 and tumor necrosis factor-alpha (TNF-α), and consequently the expansion of T helper (Th) cells. [7]. Both interleukins modulate the differentiation and proliferation of Th1 and Th17 cell subtypes [8], while TNFα is capable of enhancing the mitotic rhythm of KCs through the stimulation of cutaneous fibroblasts, production of the Keratinocyte Growth Factor (KGF) [9,10], as well as fostering leukocyte migration and T regulatory (Treg) cells suppression [11]. Specifically, the diverse role of TNFα in both the facilitation of the leukocyte migration in the cutaneous inflammation and the stimulation of KCs for the production of inflammatory cytokines has been exploited by the development of anti-TNFα drugs, displaying a high remission rate amongst PsO patients [3]. TNFα is secreted by the majority of the implicated cell types, including IFNγ-producing Th1 cells, which stimulate chemokine synthesis by the KCs [4] (Figure 1). The inflammatory cascade is amplified through the dysregulated IL-23/Th17 axis that plays a central role in the pathogenesis of the disease via the secretion of IL-17 and IL-22 [12]; IL-23 exhibits a lesional-specific increased expression profile, in contrast to other Th17-dependent inflammatory diseases, highlighting the accumulated cutaneous levels of Th17 as well as the role of IL-23 in the Th17 polarization [9]. The direct influence of IL-17 on the inflamed KCs via their increased proliferative activity is enhanced due to its secretion from the majority of the diverse cell types implicated in the disease pathogenesis, with, nevertheless, the IL-23-dependent IL-17 secretion through the activation of Th17 being widely demonstrated as a core pathogenetic mechanism and utilized as a therapeutic approach [12]. The inflammatory milieu is preserved by the abundant production of chemokines, AMPs and proinflammatory cytokines by the KCs [9] (Figure 1).

Disruption of such core mechanisms that regulate the immune response and cell proliferation is mediated through a multi-layered interaction between genetic and environmental factors. The largest genome-wide association analysis, conducted in 2017 by Tsoi et al., has uncovered 63 associated genetic loci, mapped in genes that participate in the inflammatory cascade occurring in PsO, such as the adaptive immune response and differentiation of lymphocytes [13]. Nevertheless, the complexity of PsO considering the disease’s development and progression is also attributed to environmental factors that aggravate the existing genetic predisposition. The continuous infiltration of the epidermal barricade from immunogenic stimuli, as well as smoking [14], diet [15] and sun exposure, [16] significantly alter the epigenomic profile of the diverse collection of cell types involved in the pathogenesis of PsO. Epigenetic alterations are characterized as reversible, chemical modifications in the structure of DNA without affecting the genomic sequence, thus modifying gene expression [17]. DNA methylation and post-translational histone modifications contribute to transcriptional activity, whereas post-transcriptional regulation is performed by non-coding RNA molecules (ncRNAs) [18] (Figure 1). While epigenetic changes can normally be utilized as a tool to control gene expression throughout the developmental stages of a cell type, multiple studies have associated aberrant epigenetic changes with the pathogenesis of cancer [19] and cardiovascular and autoimmune diseases [20]. This review focuses on the research conducted in characterizing the contribution of environmental factors, epigenetic factors and ncRNAs in PsO onset, in the context of KCs and the various implicated immune cell types, as well as their potential clinical relevance as biomarkers and therapeutic targeting.

## 2. Microbiome and Environmental Risk Factors

Since PsO is a multifactorial disease, genetic and environmental factors affect its onset and progression. The microbiome [21,22] is responsible for triggering adaptive and innate immune responses that extensively affect numerous immunomodulatory mechanisms (Figure 1). The connection between PsO and bacterial infections was established decades ago by providing evidence that streptococcal infection [23] can lead to PsO, and PsO patients can be distinguished from healthy individuals based on differences in their skin and gut microbiome [24]. Lifestyle factors such as diet, smoking and alcohol intake can further alter the gut microbiota composition, while metabolites derived from the latter can influence epigenetic modifying enzymes (Figure 1).

### 2.1. Skin and Gut Microbiome

Bacterial and fungal populations on the skin differ between healthy and psoriatic patients; *Streptococcus*, *Staphylococcus* and *Malassezia* [25] are increased and *Propionibacterium* and *Corynebacterium* are decreased [22]. It is suggested that *Streptococcus*’ M protein, which is highly homologous to type I keratins, can induce the expression of superantigens by T cells, further targeting KCs and causing chronic inflammation and proliferation of KCs [23]. Such superantigens are also produced by the gram-positive *Staphylococcus aureus* through the secretion of pyrogenic exotoxins, leading to severe cutaneous inflammation. HLA-DR, expressed by KCs, bind these superantigens along with secreted TNFα leading to inflammatory cascades [26]. Voluminous fungal populations of *Malassezia* spp. [27] can disrupt the epidermal barrier by producing lipases and phospholipases, attracting polymorphonuclear leukocytes and causing local skin sensitization. Production of propionate and radical oxygenase by *Propionibacterium* [26] reduces the oxidative stress levels and prevents skin inflammation. Additionally, it can modulate Th17 cells to maintain immune homeostasis. Decreased populations of *Corynebacterium* are associated with the onset and exacerbation of PsO [28], as *Corynebacterium* possesses anti-inflammatory abilities by negatively regulating interferon signaling in pDCs [28,29].

The gut microbiome is associated with skin diseases via the intestinal barrier, inflammatory mediators, and metabolites. In general, PsO patients appear to have inadequate intestinal flora [30,31], which is characterized by a reduced population of *Bacteroides* and abundant *Actinobacteria* and *Firmicutes*. *Bacteroides* possess anti-inflammatory capabilities through the production of polysaccharide-A, which is able to activate Tregs, stimulate anti-inflammatory pathways (i.e., IL-10), and thus inhibit the maintenance of inflammation. *Firmicutes* and *Bacteroides* can decrease the level of short-chain fatty acids (SCFAs) that cause inflammation and increase the vulnerability of the intestinal barrier [32]. A disrupted barrier enables microbiota dysbiosis via the circulatory system inducing both local and systemic immune responses.

### 2.2. Lifestyle

It is widely accepted that the interaction of environmental and genetic factors via epigenetic modifications contributes to the onset of a wide spectrum of diseases, including PsO (Figure 1). Food and nutrient intake can lead to alterations in the composition of the gut microbiome, allowing differential growth [33] of certain populations that are associated with PsO. In parallel with microbiota alterations, several nutrients such as sulphoraphane, curcumin [34] and omega-3 polyunsaturated fatty acids [35] can induce DNA methylation in leukocytes [36] and histone modifications by activating the epigenetic related enzymes DNMTs, HDAC and HAT. Dietary habits display a further added risk in developing PsO via the increased obesity prevalence amongst PsO cases, as described in numerous studies [37,38,39]. Meta-analysis of the leptin levels in patients with PsO confirmed the increased levels of the pro-inflammatory adipokine, a hormone that inhibits hunger and autoregulation of T cells, despite the increased between-study heterogeneity [40,41]. The implication of the adipose tissue in the inflammation through the secretion of both adipokines and the classical pro-inflammatory cytokines, such as TNFα and IL-6, establishes abdominal obesity as a risk factor for PsO, nevertheless without fully clarifying the exact causal mechanisms [42]. Smoking and alcohol can additionally enhance such psoriatic signals through a variety of mechanisms implicated in immunological disorders (i.e., KC hyperproliferation) due to the overexpression [43] of a5 integrin, cyclin D1, KGF receptor and pro-inflammatory cytokines. The epigenetic effect of tobacco is based on the induction of CpG island [44] methylation, decreased HDAC activity, increased histone methylation [45] levels and altered expression of non-coding RNAs [46,47]. 

Psychological factors as well as mood disorders, such as stress and depression, appear to play an important role in the onset and exacerbation of PsO. Stress is implicated in PsO pathogenesis through immune regulation and abnormal T cell activation. Actually, patients with PsO have lower cortisol levels [48] when stressed. Moreover, cortisol in addition to its anti-inflammatory effects, induces epigenetic changes such as DNA methylation [49], histone modifications [50], and may affect the expression of ncRNAs [51]. The psychological burden stimulates the secretion of pro-inflammatory cytokines [52], including TNFα and IL-6, further strengthening the correlation between depression and inflammatory disorders. Specifically, PsO patients undergo a significant social stigmatization due to the presence of the psoriatic plaques, leading to increased risk of social anxiety and depression [53].

UV radiation, especially UVB, is used to treat psoriatic plaques, although in some cases exposure to low light UVA may trigger photosensitivity of the skin and cause inflammation by enabling the local infiltration of neutrophiles and lymphocytes [54]. The above therapeutic mechanism of the UVB radiation is highlighted via the elevated serum levels of 25(OH) Vitamin D (the serum marker of vitamin D) in PsO patients undergoing UVB phototherapy [55]; Vitamin D binds to the Vitamin D receptor (VDR) exhibiting an immunomodulatory activity by decreasing IL-17 and IFNγ levels on Peripheral Blood Mononuclear Cells (PBMCs) [56], while Vitamin D deficiency displays a perturbated differentiation and increased proliferation of KCs [57]. Epigenetic modifications related to the Vitamin D show an anti-inflammatory and anti-proliferative profile [58], further strengthening the role of Vitamin D as an anti-inflammatory mediator.

## 3. DNA Methylation

DNA methylation is a well-studied epigenetic alteration mechanism mainly occurring in CpG islands localized on gene promoters. The addition of a methyl group into the cytosine’s C5 position, forming a 5-methylcytosine (5mC), can significantly reduce the accessibility of Transcription Factor (TF) and RNA polymerase binding sites on the DNA helix, thus repressing the transcriptional activity. DNA methylation is catalyzed through the DNA methyltransferase enzymes (DNMTs), which consist of DNMT1, DNMT3a, DNMT3b and DNMT3L. DNMT1 prefers hemimethylated DNA and is characterized as a “maintenance DNMT” due to its repairing activity, while DNMT3L induces the de novo methyl group transfer activities catalyzed by both DNMT3a and DNMT3b [59,60]. Demethylation of 5mC is performed either from the ten-eleven translocation (TET) enzymes with the formation of the intermediate 5 hydroxyl-methylated cytosine (5hmC), or by the deamination of 5mC and the utilization of the base excision repair (BER) pathway [60].

### 3.1. DNA Methylation in KCs

The role of KCs in PsO pathogenesis includes both the formation of the psoriatic plaques due to their increased proliferation and the maintenance of inflammation through their contribution in the inflammatory milieu and the production of multiple proinflammatory cytokines [61]. Thus, epigenetic modifications affecting the transcriptional activity of genes involved in these pathways may contribute to PsO pathogenesis. 

The pro-inflammatory Ca^2+^ binding proteins S100A8 and S100A9 are members of the S100 family. The heterodimeric protein S100A8/A9 is released actively at the time of inflammation, modulating its progression by stimulating leukocyte recruitment and inducing cytokine secretion [62]. These molecules are highly up-regulated in KCs and leukocytes of psoriatic skin and their expression is induced by IL-10 in differentiated human dendritic cells [63]. The methylation status of their genes’ promoter has been characterized by multiple whole-genome methylation analyses (Figure 1).

Roberson et al. were the first to observe global alterations of CpG methylation in skin from PsO patients compared to skin from healthy volunteers. They identified 674 hypermethylated and 444 hypomethylated CpG sites, which were mainly localized on gene promoters, unveiling significant correlation between methylation and the expression of nearby genes such as *C10orf99*, *OAS2* and *KYNU* [64] (Figure 1). Furthermore, the methylation patterns were shown to be reversible to a non-psoriatic state after the administration of anti-TNFα therapy for one month. In the study by Zhang et al., whole-genome DNA methylation of psoriatic and non-psoriatic skin samples showed more hypermethylated regions (15,684) than hypomethylated (11,084). *PDCD5* and *TIMP2*, which induce KC proliferation, were hypermethylated and hypomethylated, respectively, exhibiting reversed expression levels [65]. Chandra et al. reported that 25% of differentially methylated CpGs were located at characterized PsO susceptibility (PSORS) loci, including PSORS2, PSORS4, PSORS6 and PSORS7, encoding several genes such as *S100A9*, *SELENBP1, CARD14, KAZN* and *PTPN22* with an inverse correlation between methylation and expression (Figure 1). Differentially methylated genes associated with histopathological aspects were also found, including *AIF1*, *FFAR2* and *TREM1*, which are implicated in neutrophil and leukocyte chemotactic events [66]. Hou et al. detected 96 hypermethylated genes, including MAPK signaling-related genes, such as *CACNA2D3* and *SRF*, and 234 hypomethylated genes participating in the increased angiogenesis of psoriatic lesion, namely, *NRP2, VEGF,* and *VASH1* [67]. In PsO skin, Zhou et al. discovered nine differentially methylated sites near metabolism-related genes, including *CYP2S1*, *ECE1, EIF2C2*, *MAN1C1*, and *DLGAP4*, whose methylation was negatively correlated with their expression. In the intergenic area surrounding *CYP21*, considerably low methylation has been observed [68].

There is ample evidence in the literature linking the methylation status and expression level of genes that are candidates for PsO pathogenesis. Bisulfite sequencing in skin lesions revealed that hypermethylation of *p14^ARF^* promoter resulted in its downregulation [69]. The low expression of *SFRP4* in psoriatic skin, which is involved in the Wnt pathway and KC’s hyperproliferation, is correlated with its promoter’s hypermethylation [70] (Figure 1). The Wnt pathway plays a key role in PsO by regulating the proliferation and differentiation of KCs [71]. Additional evidence exists regarding the non-malignant effect of specialized promoters’ methylation status in a human malady. The promoter of the SHP-1 isoform was found to be hypomethylated in psoriatic lesions, indicating that the methylation of *SHP-1*’s promoter in PsO might be related to the STAT3’s binding affinity, due to the upregulation of the latter in the lesional skin [72] (Figure 1). The same group also studied ID4, a protein that participates in cell proliferation, differentiation and apoptosis, as well as in tumorigenesis (cholangiocarcinoma, breast cancer, lymphoma). *ID4* promoter hypermethylation promoter was linked with parakeratosis, which refers to deficient development of KCs, and skin-related cellular differentiation in PsO cases [73] (Figure 1). The promoter of the *p16^INK4a^* gene, which is involved in hyperproliferative skin diseases, was found to be hypermethylated in psoriatic skin [74]. Sheng et al. investigated the hypomethylation of *CYP2S1* and further identified the hypomethylation of two extra loci within the *CYP2S1* region, leading to its upregulation in psoriatic tissues [75] (Figure 1). Members of the growth arrest and DNA damage-inducible gene family, such as *GADD45a* and *GADD45b*, exhibit low expression in psoriatic lesional skin [76]. Specifically the expression *GADD45a*, which has a demethylase activity, was found to be positively correlated with IFN-γ and TNFα expression. Its depletion leads to hypermethylation of *UCHL1* promoter in PsO cases [77]. The expression of *WIF1*, an inhibitor of Wnt signaling, is linked to its promoter’s demethylation, which is a result of *DNMT1* silencing, while its hypermethylation is a consequence of *DNMT1* overexpression. Indirubin, a traditional medicine utilized for treating various inflammatory diseases, inhibits the expression of *DNMT1* and the methylation of *WIF1* promoter, as well as the expression of Wnt-pathway core genes, such as *FZD2*, *FZD5*, and β-catenin [78].

### 3.2. DNA Methylation in Immune Cells

Despite the predominant role of KCs in the pathogenesis of cutaneous diseases, PsO, as an autoimmune disorder, is driven by the substantial activation of multiple immune cell types, which further stimulate KCs’ proliferation through the secretion of pro-inflammatory cytokines. Moreover, PsO is considered to be a T-cell-mediated autoimmune disease, since the role of Th cells, as well as their secretome, has been extensively studied and targeted therapeutically [79]. Immune cells can be isolated either from lesional skin or PBMCs. The latter is a non-invasive approach and has therefore been established as the standard method for studying PsO-related immune cells.

PBMCs from psoriatic patients have been shown to exhibit aberrant DNA methylation, for example, hypermethylation of *p14^ARF^*, *MBD2*, *MeCP2* and hypomethylation of *DNMT1* [69] (Figure 1). Genome-wide DNA methylation profiling of CD4^+^ T cells unveiled the significantly hypermethylated promoters of immune-related X chromosome genes, such as *SLITRK4*, *EMD*, *ZIC3*, *CXorf40A*, *HDAC6*, *IKBKG*, *SH3KBP1*, *OTUD5*, *NDUFA1*, *WNK3* and *MSL3* [80]. Recent studies uncovered the hypermethylated profile of genes that are implicated in the TGFβ pathway, including *SNX25, STAD3* and *BRG1*, from whole blood samples of monozygotic twins [81] (Figure 1). Additional comparison of CD8^+^ T cells in monozygotic twins from psoriatic samples and healthy controls identified 110 hypermethylated and 224 hypomethylated loci. DNA methylation analyses of CD8^+^ T cells between PsO, psoriatic arthritis cases and healthy controls revealed numerous differences, indicating that DNA methylation screening in these cell subtypes could act as a potential diagnostic biomarker [82]. Furthermore, genome-wide DNA methylation profiling from peripheral whole blood displayed that *FOXP3* is hypermethylated, leading to reduced T_reg_ levels in patients with PsO [83] (Figure 1).

Bisulfite-sequencing on a targeted gene panel revealed the low methylation levels of *p15* and *p21* promoters in hematopoietic stem cells; *p15*, *p21* and *p16* exhibit similar methylation patterns and play a well-established role in controlling cell cycle [84,85] (Figure 1). Research conducted in CD4^+^ T cells of monozygotic twins with PsO showed the promoter hypomethylation of transcription-regulator *ZNF99* gene. In CD8^+^ T cells associated with PsO, hypomethylation of the serine/threonine MAST3 and MTOR kinases, and hypermethylation of the *PM20D1* peptidase gene was shown [86] (Figure 1).

## 4. Histone Modifications

The post-translational histone modification (PTM) process is an important mechanism of gene expression regulation since these proteins directly participate in DNA organization and accessibility. Briefly, nucleosomes are the fundamental subunit of chromatin and are composed of histone octamers. Each histone (H2A, H2B, H3 and H4) is represented twice in the nucleosome structure that forms a binding scaffold for 147 DNA base pairs [87]. PTMs usually occur in the overhanging N-terminal tails of histones, resulting in either enhanced transcriptional activity through nucleosome unwinding and euchromatin formation, or strengthened DNA-histone interactions that form heterochromatin and suppress gene expression. Several types of histone modifications, implicated in both gene silencing and enhanced transcription, have been described, including adenylation, methylation, phosphorylation, ADP ribosylation and sumoylation among others [88].

### 4.1. Histone Modifications in KCs

Epigenetic regulation of KCs is an essential part of chronic skin inflammation. Recent research demonstrated that decreased H3K9 dimethylation leads to increased IL-23 expression in KCs; H3K9me2 levels play a key role in regulating basal and TNF-induced IL-23A expression [89]. H3K27me3 and *EZH2*, a histone methyl-transferase enzyme, were significantly enriched in cutaneous biopsies from individuals with PsO when compared to healthy controls. *EZH2* is implicated in cell proliferation and tumorigenesis [90] and thus its transcriptional silencing affects the proliferation and differentiation of KCs.

Sirtuin (SIRT) is a family of (NAD^+^)-dependent deacetylases, involved in cell apoptosis, gene transcription, tumor development, autoimmune inflammation and epigenetic modification processes. Specifically, *SIRT1* regulates inflammation-associated signaling pathways [91,92]. Hwang et al. showed that *HDAC-1* is overexpressed and *SIRT1* displays a decreased expression in skin biopsies of patients with PsO [93]. GLS1-mediated glutaminolysis induces proliferation of KCs in PsO and promotes Th17 and γδ T17 cell differentiation through the acetylation of H3 on Il17a promoter [94]. The first whole-genome study for histone modifications showed that H3K27 is hyperacetylated in 60% of the overexpressed gene promoters in cutaneous lesions and binding sites of overexpressed TFs in lesional skin, such as *GRHL* [95] (Figure 1). *WT1*, a TF implicated in cell proliferation and apoptosis, is highly expressed in psoriatic skin lesions and has two binding sites in the IL-1β gene promoter. IL-1β is produced by KCs, with its gene promoter showing considerably high histone acetylation levels that positively correlate with histone acetyltransferases p300 (P300) expression [96]. H3K27 hyperacetylation of *RPL22* promoter in PsO lesional skin leads to overexpression of *RPL22*, which is linked to CyclinD1 upregulation, inducing KC proliferation. *RLP22* also prevents KC apoptosis and is involved in CD4^+^ T cell chemotaxis [97].

### 4.2. Histone Modifications in Immune Cells

PBMCs from PsO patients exhibit higher H3K4 methylation levels when compared to healthy individuals. Responders and non-responders in biological therapies tend to have different H3K27 and H3K4 methylation profiles [98] (Figure 1). Regulation of Th17 cell differentiation can be achieved by TCR-induced H3K27 demethylase Jmjd3, which is overexpressed and decreases H3K27me3 levels. Jmjd3 controls chromatin accessibility of numerous Th17-related loci, such as Th17-specific gene promoters, and induces Th17 cell differentiation by decreasing H3K27me3 enrichment [99]. Zhang et al. unveiled decreased H4 acetylation in psoriatic PBMCs, downregulation of *P300*, *CBP* and *SIRT1* as well as increased HDAC1 levels [100] (Figure 1).

## 5. Non-Coding RNAs

Non-coding RNAs (ncRNAs) are RNA molecules that are not translated into functional proteins. They are typically grouped into distinct families according to their size and function. Most ncRNA families play a key role in directly or indirectly affecting gene expression at the transcriptional and post-transcriptional level. The microRNA (miRNA) family consists of small (~18–23 nucleotides), single-stranded RNA molecules that are loaded on and guide the RNA-induced silencing complex (RISC) to mediate mRNA degradation and/or translation suppression [101]. Circular RNAs (cirRNAs) form a continuous loop through their linkage on the 5′ and 3′ termini, establishing them as stable, exonuclease-proof RNA molecules with numerous roles in transcriptional and translational regulation [102]. Long non-coding RNAs (lncRNAs) are defined as longer than 200 nucleotides transcripts with an emerging role in the pathogenesis of autoimmune diseases [91]. LncRNAs and circRNAs can regulate gene expression by participating in processes that alter chromatic conformation, forming triplexes with DNA as well as interfering with transcription enzymes [103].

Due to the abundance of the ncRNAs implicated and studied in the context of PsO, we conducted an exhaustive literature search regarding the differential expression of ncRNAs in cutaneous biopsies, serum levels as well as PBMCs. We filtered the screened studies according to the importance of the ncRNAs under study, evaluated through their statistically significant differential expression in contrast to healthy controls as well as their identified, direct or indirect target genes. Non-coding RNAs screened by more than one study were also identified as important regulators of the psoriatic transcriptome.

### 5.1. MiRNAs in KCs

The immortalized nontumorigenic human epidermal (HaCaT) cell line and KCs have been widely used in PsO studies as they are easy to isolate and provide reliable results regarding the transcriptome and proteome profile of the disease [104]. Fibronectin 1 (*FN1*) and integrin subunit α9 (*ITGA9*) signaling pathways are both implicated in cell motility and direct targets of miR-4516, which was found to be downregulated in cutaneous PsO biopsies (Figure 1). Specifically, Chowdhari et al. found significant overexpression of both *FN1 and ITGA9* as well as STAT3 in PsO, which could be partly responsible for the KCs activated state in lesional skin since it induces proliferation and terminal differentiation [105]. MiR-424 has been also investigated as a potential biomarker in PsO given its regulatory role in signaling pathways that orchestrate differentiation and cell cycle regulation in KCs [106] (Figure 1). The decreased miR-424 expression has been associated with the overexpression of *MEK1* and *CCNE1*, members of the metabolic pathways responsible for the abnormal KC proliferation observed in the clinical manifestation of the disease; however, the exact regulatory mechanism has yet to be defined. Another miRNA whose down-regulation has been associated with PsO is miR-145, a molecule that has been widely studied in immune-mediated inflammatory diseases due to its inhibitory role in cell proliferation and immune responses [107] (Figure 1). It has been observed that miR-145 downregulation promotes proliferation and chemokine expression in the lesional skin, as it directly targets *MLK3*, which in turn regulates STAT3 and NF-κB TFs. Increased expression of miR-21 has been associated with the epidermal downregulation of *TIMP-3*, leading to the activation of *TACE*, which subsequently induces TNFα overexpression and a psoriasis-like phenotype [108] (Figure 1). 

MiR-200c, a miRNA involved in apoptosis and senescence of KCs, was found to be upregulated in lesional skin compared to non-lesional skin biopsies and healthy controls [109] (Figure 1). MiR-200c is also known to directly repress *SIRT1*, which has a key role in oxidative stress and the regulation of skin inflammation, as well as *eNOS* and *FOXO1,* which have a significant role in regulating the function and preservation of endothelial cells. MiR-200c expression also shows a positive correlation, without being a direct regulator of molecules involved in inflammation such as *IL-6* and *COX-2*, and plaque destabilization such as *MMP-1* and *MMP-9*. Table 1 presents further deregulated miRNAs in KCs from PsO patients.

### 5.2. MiRNAs in Immune Cells and Serum

Fu et al. showed that miR-138, whose expression levels regulate the balance between Th1 and Th2 cells by targeting RUNX3, was found downregulated in PBMCs of Pso patients [125] (Figure 1). RUNX3 is an important TF regulating cell proliferation and apoptosis, and its increased expression in PsO classifies it as a key gene for PsO susceptibility. MiR-143 downregulation exhibits a significant correlation with PsO severity; specifically, patients with stable disease stages showed higher miR-143 expression levels, while patients in progressive stages had lower expression levels [126] (Figure 1). *BCL2*, which is thought to be responsible for shortening the lifetime of cortical cells, has been proven a direct target of miR-143 [126]. Additionally, García-Rodríguez et al. showed that the upregulation of miR-155 in PsO plasma samples is an important regulator of *SOCS1*, a susceptibility locus of PsO [127]. The miRNA-155/SOCS1 pathway is targeted in macrophages by Vitamin D or Vitamin D Receptor (VDR) signaling to reduce the inflammatory response [128] (Figure 1). The upregulation of miR-210 in CD4^+^ T cells has also been found to significantly correlate with PsO onset and progression, mainly by targeting *FOXP3* [129] (Figure 1). *FOXP3* displays a central role in the development and diverse functionality of Treg cells, as it appears to facilitate their differentiation through genetic programming [129], thus establishing it as an important contributor to the pathogenesis of the disease. In normal CD4^+^ T cells, overexpression of miR-210 can indirectly, contribute to the expression of inflammatory cytokines such as IFN-γ and IL-17 while suppressing other cytokines such as IL-10 and TGF-β, which are secreted by Tregs. Table 2 presents further deregulated miRNAs in PBMCs and plasma from PsO patients.

### 5.3. LncRNAs in PsO

Despite the established regulatory role of lncRNAs, there is still limited evidence regarding their participation in PsO pathogenesis. The maternally expressed gene 3 (*MEG3*), a downregulated lncRNA in HaCaT cells, has an identified miR-21 binding site, thus acting as a sponge or decoy for miR-21. It is postulated that *MEG3* participates in the regulation of PsO KCs proliferation and apoptosis as well as the expression of *CASP8* through its interplay with miR-21 [135]. By utilizing a luciferase reporter assay, it was confirmed that MSX2P1, a lncRNA overexpressed in HaCaT and KCs, is a direct target of miR-6731, thus negatively affecting its function on other RNAs. Further research in IL-22-stimulated KCs revealed MSX2P1’s indirect role in increasing the protein levels of S100A7, IL-23, NF-κB, TNFα, IL-12β, HLA-C, and CCHCR [136]. Psoriasis-susceptibility-related RNA Gene Induced by Stress (PRINS) [137] and GAS5 [138] are lncRNAs displaying an under- and over-expression pattern, respectively, in the serum levels of patients with PsO. Specifically, plasma levels of PRINS transcripts were found to be down-regulated in patients with PsO, similar to its direct target *G1P3* and interacting partner *NPM*, while PRINS’ miRNA targets that function as decoys, consisting of miR-124, miR-203a, miR-129, miR-146a and miR-9, were overexpressed, thus indicating a possible lncRNA–miRNA–mRNA axis of diagnostic value.

While the role of circRNAs in PsO progression remains obscure, CDR1as, a ciRNA significantly downregulated in cutaneous PsO biopsies, has been associated with numerous genes that are involved in the pathogenesis of the disease, such as *EGR3, GATA6, GATA3* and *FOXN3* [139]. However, the direct regulatory mechanism remains unclear (Figure 1). Xiaoxin Liu et al. also showed that circRNA hsa_skin_088763 was down-regulated in lesional skin compared to normal controls. It is postulated that this circRNA is indirectly associated with several PsO-related genes such as *GATA6*, *SIK2*, *IL17RD*, *EGR3*, *FAS*, *LRIG1*, and *PPARGC1A*, due to their shared regulatory ncRNAs (Figure 1), thus characterizing it as a competing endogenous RNA (ceRNA). A comprehensive list of all deregulated lncRNAs is presented in Table 3 and Table 4, stratified based on the cell type under study.

## 6. Therapeutic Approaches Targeting the Epigenetic Mechanisms

In recent years, biologic drugs targeting TNF, IL-23 and IL-17 and small-molecule drugs such as phosphodiesterase-4 (apremilast) and Janus kinase (JAK) inhibitors have been effective in plaque psoriasis clinical management. The predominant role of epigenetic modifications in the pathogenesis of complex diseases, as analyzed in the framework of PsO, establishes the therapeutic interventions targeting the epigenome, a promising clinical field. Numerous inhibitors of molecules that participate in the epigenetic reprogramming, including DNMTs and HDAC, have been extensively utilized in clinical trials. Recently FDA-approved agents, combined with cytotoxic chemotherapies, show promising results despite their limited implementation, mostly on hematologic malignancies [146]. Such repurposing approaches, already applicable in cancer, might prove beneficial in PsO considering the diverse cell subtypes that are involved in its pathogenesis. 

Reservatol, a polyphenol with anti-inflammatory properties, was shown to stimulate the expression of *SIRT1* leading HaCaT cells to death [147]. Recently, trichostatin A (TSA), a class I and II HDAC inhibitor (HDACi), significantly decreased KC’s proliferative phenotype, both in vitro and in vivo [148]. These results are in accordance with previous studies examining the effect of TSA on human Tregs and the prevention of their differentiation into IL-17A producing cells through the overexpression of *FOXP3* [149,150]. Another example of an epigenetic-driven therapeutic intervention is peroxisome proliferator-activated receptor gamma (PPARγ) and/or alpha (PPARα) antagonists, which inhibit *AQP3* expression in KCs, while agonists induce the differentiation of the latter, establishing them as a topical treatment for cutaneous diseases such as PsO. AQP3 is a water channel protein that regulates multiple aspects of KCs [151,152], with its expression induced by HDACs, particularly HDAC3, via acetylated transcription factors such as the family of p53 and PPARs.

MiRNA-mediated gene expression regulation is another approach in the anti-PsO therapeutic arsenal. MiRNAs exhibit a diverse role through their interaction with numerous transcripts and can therefore affect multiple pathways implicated in PsO. Imiquimod-induced psoriasis-like murine models were treated with a miR-210 antisense molecule-containing topical gel. Based on the predominant role of miR-210 in the regulation of multiple genes expressed in CD4^+^ T cells, as analyzed before, cellular markers of cell proliferation were significantly decreased in KCs, while the imbalance of CD4^+^ T cells was reversed to a non-pathological state [153]. Another topical-application approach referred to the usage of quaternized starch (Q-starch) as a miRNA-197 delivery system in lesional-xenotransplantated mice, alongside ultrasound for increased cutaneous permeability. The Q-starch/miRNA-197 complex was able to alleviate the psoriatic symptoms through the targeting of IL-22RA1 and IL-17RA transcripts, nevertheless without a homogeneous effect along the transplanted psoriatic skin sample [154]. Locked nucleid acid (LNA) *anti-miR-21* oligonucleotides were also assessed for their efficacy as a therapeutic approach in a double-knockout PsO murine model, displaying an important amelioration in the histopathological symptoms of the disease [108], further highlighting the role of miR-21 overexpression in PsO. Additionally, Qiao et al. inhibited miR-6731 in IL-22-stimulated HaCaT cells, showing an increased proliferative activity of the IL-22-induced KCs, while protein expression levels of therapeutic targets of PsO, including TNFα, IL-23, HLA-C as well as inflammatory molecules such as NF-κB and the PSORS1 locus were significantly overexpressed. These results imply a protective role of miR-21 in the pathogenesis of PsO [135].

## 7. Discussion

The etiopathology of PsO lies in the complex interactions between genetic, immunological and environmental factors, including but not limited to the imbalance in the gut and skin microbiome, as well as lifestyle and stress-inducing factors. The effect of genome–environment interactions can extend to the epigenome with direct and indirect modulation of DNA methylation and histone modifications in PsO-related loci. These cascading effects are further amplified by the function of ncRNAs, which in numerous ways regulate the expression of PsO-associated genes and pathways. However, epigenetic modifications and deregulation of ncRNA molecules occur in the spectrum of both KCs and immune cells, while the polygenicity of PsO aggravates candidate-gene approaches, thus obscuring the characterization of the exact mechanisms that alter the disease predisposition in individuals. Furthermore, skin tissue and immune cells consist of abundant and heterogeneous cellular populations, where each cell type exhibits a distinct epigenomic and, thus, transcriptomic profile. This diversity dramatically increases the complexity of research conducted in the field. The secretome of implicated cell types is affected by the distinct epigenetic and regulatory profiles of each sub-population, which is triggered by stress-inducing factors and the disease progression (Figure 1). Nevertheless, with the advent of next-generation sequencing, modern flow cytometry techniques and genome-wide analyses, the epigenetic reprogramming that participates in homeostasis disruption and the cutaneous inflammatory cascade is gradually elucidated, leading to the clarification of PsO pathogenesis. 

Even though the epigenetic modifications cannot be utilized as clinical biomarkers for the disease progression, shedding light upon the molecular mechanisms governing their development and maintenance can potentially uncover novel therapeutic targets associated with the induced epigenetic changes. In contrast, ncRNAs and especially miRNAs have a rich history of being used as disease biomarkers and targets of therapeutic intervention, despite the difficulties imposed by the off-target effects due to the wide spectrum of pathways affected by miRNAs. 

Future studies should focus on developing methods for reversing DNA methylation in the context of PsO therapeutic interventions, as a way to understand the molecular discrepancies between responders and non-responders to therapy. Additionally, the catalogue of ncRNAs implicated in PsO should be significantly enriched to provide an extensive view of the targeted genes set and an accurate description of the vast interactome that underlies PsO. The gene regulatory networks that will emerge from the combination of epigenetic and ncRNA meta-analyses, as well as the genetic predisposition to PsO, will facilitate the development of a new generation of highly precise therapeutic approaches with minimum adverse effects and maximum impact.

## Figures and Tables

**Table 1:** Deregulated ncRNAs in PsO in keratinocytes.

Author, Year	ncRNA	Expression	Direct Targets (Indirectly Affected Genes)	Cell Type
Yan et al., 2018 [107]	miR-145-5p	Down	*MLK3*, (*NF-κB*, *STAT3*, *AKT*, *GSK*, *MAPK*, *MTOR*)	HEKs
Chowdhari et al., 2017 [105]	miR-4516	Down	*FN1, ITG9, STAT3*	HaCaT
Ichihara et al., 2011 [106]	miR-424	Down	(*MEK1*, *CCNE1*)	HEKs
Huang et al., 2021 [110]	miR-193b-3	Down	*ERBB4*, (*STAT3*, *NF-κB*, *IL-6*, *CXCL1*, *CCL20*, *BD-2*)	HaCaT
Pan et al., 2019 [111]	miR-125b	Down	*BRD4,* (*JAG1)*	HaCaT
Xu et al., 2011 [112]	miR-125b	Down	*FGFR2*	HEKs
Yu et al., 2017 [113]	miR-194	Down	*GRHL2*, *SOX5*	HEKs
Rongna et al., 2018 [114]	miR-876-5p	Down	*ANGPT1*	HaCaT
Zheng et al., 2019 [115]	miR-181b-5p	Down	*AKT3*	HEKs
Zheng et al., 2019 [115]	miR-125b-5	Down	*AKT3*	HEKs
Jiang et al., 2017 [116]	miR-486-3p	Down	*K17*	HaCaT
Zhao et al., 2022 [117]	miR-214-3p	Down	*FOXM1*	HaCaT
Yan et al., 2015 [118]	miR-31	Up	*PPP6C*	HEKs
Guinea-Viniegra et al., 2014 [108]	miR-21	Up	*TIMP3*	HEKs
Zhang et al., 2018 [119]	miR-17-92	Up	*CDKN2B, SOCS1*	HEKs
Zhang et al., 2020 [120]	miR-142-3p	Up	*SEMA3A*	HaCaT
Xu et al., 2017 [121]	miR-155	Up	(PTEN, PIP3, AKT, BAX, Bcl-2)	HaCaT
Wang et al., 2019 [122]	miR-223	Up	*PTEN*	HaCaT
Sonkoly et al., 2007 [123]	miR-203	Up	*SOCS3*, (*STAT3*)	HEKs
Zibert et al., 2010 [124]	miR-221, miR-222	Up	(*TIMP3*)	HEKs

Abbreviations: ncRNA, non-coding RNA; miR, microRNA; HEK, human embryonic kidney cell line; HaCaT, human epidermal keratinocyte cell line.

**Table 2:** Deregulated ncRNAs in PsO in immune cells and plasma.

Author, Year	ncRNA	Expression	Direct Targets (Indirectly Affected Genes)	Cell Type
Immune cells				
Zheng et al., 2017 [126]	miR-143	Down	*BCL2*	PBMCs
Fu et al., 2015 [125]	miR-138	Down	*RUNX3*	CD4^+^ T cells
Garcıa–Rodrıguez et al., 2016 [128]	miR-146a	Up	*TRAF6*, *IRAK1*, (*NF-κΒ*, *IL-6*, *TNFα*)	PBMCs
Garcıa–Rodrıguez et al., 2016 [128]	miR-21	Up	*PDCD4*	PBMCs
Garcıa–Rodrıguez et al., 2016 [128]	miR-155	Up	*SOCS1*	PBMCs
Serum levels				
Duan et al., 2019 [130]	miR-126	Down	*-*	Plasma
Ichihara et al., 2011 [106]	miR-424	Down	(*MEK1, CCNE1*)	Plasma
Zhao et al., 2014 [129]	miR-210	Up	*FOXP3,* (*IFN-γ*, *IL-17, IL-10, TGF-β*)	Plasma
Magenta et al., 2019 [109]	miR-200c	Up	*SIRT1*, *eNOS*, *FOXO1*	Plasma
Wang et al., 2017 [131]	miR-200a	Up	*-*	Plasma
Borska et al., 2017 [132]	miR-31	Up	*-*	Plasma
García-Rodríguez et al., 2014 [133]	miR-33	Up	*ABCA1*	Plasma
Guo et al., 2013 [134]	miR-369-3p	Up	-	Plasma

Abbreviations: ncRNA, non-coding RNA; miR, microRNA; PBMCs, peripheral blood mononuclear cells.

**Table 3:** Deregulated lncRNAs in psoriasis in keratinocytes. In this table, miRNAs, which are targets of lncRNAs that function as decoys, are highlighted in bold.

Author, Year	Non-Coding RNA	Expression	Target Genes (Indirect Targets)	Cell Type
Liu et al., 2021 [139]	CDR1as	Down	(*EGR3*, *GATA6*, *GATA3*, *FOXN3*)	HEKs
Liu et al., 2021 [139]	hsa_skin_088763	Down	(*GATA6*, *SIK2*, *IL17RD*, *EGR3*, *FAS*, *LRIG1*, *PPARGC1A*)	HEKs
Jia et al., 2019 [135]	MEG3	Down	**miR-21**	HaCaT
Liu et al., 2021 [139]	hsa_skin_05227	Down	*GATA6*	HEKs
Yazıcı et al., 2021 [140]	7SL-RNA	Down	*-*	HEKs
Moldovan et al., 2020 [141]	circEXOC6B	Down	*-*	HEKs
Moldovan et al., 2020 [142]	circSLC8A	Down	*-*	HEKs
Moldovan et al., 2020 [142]	circRHOBTB	Down	*-*	HEKs
Qiao et al., 2018 [136]	MSX2P1	Up	**miR-6731**, (*S100A7*)	HaCaT, NHEK
Gao et al., 2018 [142]	MIR31HG	Up	-	HaCaT
Li et al., 2017 [143]	lncRNA-H19	Up	**miR-130b**, (*DSG1*)	HEKs
Szegedi et al., 2010 [144]	PRINS	Up	-	HeLa
Cai et al., 2019 [145]	PRANCR	Up	-	HEKs
Moldovan et al., 2020 [141]	cTNFRSF21,	Up	-	HEKs
Moldovan et al., 2020 [141]	circDOCK1	Up	-	HEKs
Moldovan et al., 2020 [141]	circARAP2	Up	-	HEKs
Moldovan et al., 2020 [141]	circDDX21	Up	-	HEKs
Moldovan et al., 2020 [141]	circZRANB1	Up	-	HEKs

Abbreviations: circRNA, circular RNA; lncRNA, long non-coding RNA; HEKs, human embryonic kidney cell line; HaCaT, human epidermal keratinocyte cell line; NHEK, normal human epidermal keratinocyte.

**Table 4:** Deregulated lncRNAs in psoriasis in serum. In this table, miRNAs, which are targets of lncRNAs that function as decoys, are highlighted in bold.

Author, Year	Non-Coding RNA	Expression	Target Genes (Indirect Targets)	Cell Type
Abdallah et al., 2022 [137]	PRINS	Down	**miR-124**, **miR-203a**, **miR-129**, **miR-146a**, **miR-9**, *GIP3*	Plasma
Shehata et al., 2021 [138]	GAS5	Up	*-*	Plasma

## Data Availability

The datasets generated or analyzed during the current study are available from the corresponding author on reasonable request.
